# An Economic Evaluation of Neonatal Screening for Inborn Errors of Metabolism Using Tandem Mass Spectrometry in Thailand

**DOI:** 10.1371/journal.pone.0134782

**Published:** 2015-08-10

**Authors:** Kittiphong Thiboonboon, Pattara Leelahavarong, Duangrurdee Wattanasirichaigoon, Nithiwat Vatanavicharn, Pornswan Wasant, Vorasuk Shotelersuk, Suthipong Pangkanon, Chulaluck Kuptanon, Sumonta Chaisomchit, Yot Teerawattananon

**Affiliations:** 1 Health Intervention and Technology Assessment Program (HITAP), Ministry of Public Health, Nonthaburi, Thailand; 2 Division of Medical Genetics, Department of Pediatrics, Faculty of Medicine Ramathibodi Hospital, Mahidol University, Bangkok, Thailand; 3 Division of Medical Genetics, Department of Pediatrics, Faculty of Medicine Siriraj Hospital, Mahidol University, Bangkok, Thailand; 4 Center of Excellence for Medical Genetics, Department of Pediatrics, Faculty of Medicine, Chulalongkorn University, Bangkok, Thailand; 5 Genetic Unit, Department of Pediatrics, The Queen Sirikit National Institute of Child Health, Department of Medical Services, Ministry of Public Health, Bangkok, Thailand; 6 Neonatal Screening Operation Centre, Department of Medical Science, Ministry of Public Health, Nonthaburi, Thailand; Deakin University, AUSTRALIA

## Abstract

**Background:**

Inborn errors of metabolism (IEM) are a rare group of genetic diseases which can lead to several serious long-term complications in newborns. In order to address these issues as early as possible, a process called tandem mass spectrometry (MS/MS) can be used as it allows for rapid and simultaneous detection of the diseases. This analysis was performed to determine whether newborn screening by MS/MS is cost-effective in Thailand.

**Method:**

A cost-utility analysis comprising a decision-tree and Markov model was used to estimate the cost in Thai baht (THB) and health outcomes in life-years (LYs) and quality-adjusted life year (QALYs) presented as an incremental cost-effectiveness ratio (ICER). The results were also adjusted to international dollars (I$) using purchasing power parities (PPP) (1 I$ = 17.79 THB for the year 2013). The comparisons were between 1) an expanded neonatal screening programme using MS/MS screening for six prioritised diseases: phenylketonuria (PKU); isovaleric acidemia (IVA); methylmalonic acidemia (MMA); propionic acidemia (PA); maple syrup urine disease (MSUD); and multiple carboxylase deficiency (MCD); and 2) the current practice that is existing PKU screening. A comparison of the outcome and cost of treatment before and after clinical presentations were also analysed to illustrate the potential benefit of early treatment for affected children. A budget impact analysis was conducted to illustrate the cost of implementing the programme for 10 years.

**Results:**

The ICER of neonatal screening using MS/MS amounted to 1,043,331 THB per QALY gained (58,647 I$ per QALY gained). The potential benefits of early detection compared with late detection yielded significant results for PKU, IVA, MSUD, and MCD patients. The budget impact analysis indicated that the implementation cost of the programme was expected at approximately 2,700 million THB (152 million I$) over 10 years.

**Conclusion:**

At the current ceiling threshold, neonatal screening using MS/MS in the Thai context is not cost-effective. However, the treatment of patients who were detected early for PKU, IVA, MSUD, and MCD, are considered favourable. The budget impact analysis suggests that the implementation of the programme will incur considerable expenses under limited resources. A long-term epidemiological study on the incidence of IEM in Thailand is strongly recommended to ascertain the magnitude of problem.

## Introduction

Inborn errors of metabolism (IEM) comprise more than 30 genetic disorders that can lead to several serious long-term complications to neonatal and young children [1]. Without rapid diagnosis and appropriate treatment, these diseases can cause mental retardation (MR), physical disabilities, and even death [2]. Although the incidence of IEM seems to be low and varied among different ethnicities [3], high incidences are found in the North American and European populations with an incidence of 40.00 and 29.51 cases per 100,000 live births respectively [4, 5] whereas the incidences of IEM in Asian populations range between 16.08–26.35 in 100,000 live births [6–8].

Tandem mass spectrometry (MS/MS) is an advanced technology that has the ability to identify more than 30 diseases [9] by testing compounds from a single dried blood sample collected from infants during their second to third days of life [10]. Analysis for identifying each condition is simultaneous and rapid with high specific sensitivity (100%) and specificity (100%) [11]. With this technology, many high- income countries such as Italy, Denmark, Canada, Australia, Qatar, and Taiwan have expanded their neonatal screening programme in order to cover more IEM, which in the past consisted of only phenylketonuria (PKU) [12].

In Thailand, a neonatal screening programme was introduced in 1996 to screen for PKU [13]. Currently, PKU is the only disease screened among the IEM group and the screening method used is the Guthrie test due to its simplicity and inexpensiveness. For other diseases, to date, no study has been carried out to identify the magnitude of the problem especially in terms of incidence and/or prevalence of the diseases in a systematic way. Without such fundamental information to support the necessity of advanced and expensive technology, convincing policy-makers to introduce MS/MS as a population-based screening tool in Thailand will be very challenging.

So far, many studies have shown that MS/MS is cost-effective in their specific country settings [14–22]. However, due to generalizability and transferability issues, the results of the economic evaluations in the original country of study cannot be transferred to other countries because of the differences in multiple factors (e.g. demography, epidemiology of disease, health infrastructure, clinical practice, and healthcare cost) [23–25]. Thus, this study was conducted to determine the cost-effectiveness of screening and treatment for selected IEM in Thailand. The result of this study will be mainly used to support policy-makers of the National Health Security Office (NHSO) to determine whether this screening intervention should be included into the benefits package of the country’s Universal Health Coverage (UHC) scheme.

## Materials and Methods

### Selection of IEM disorders

By recognising that screening all diseases detectable by MS/MS may not be possible in Thailand where healthcare resources are limited. For example, with a small number of physicians who specialise in IEM treatment, managing medical care for all detected patients is impracticable. Therefore, only most significant diseases will be screened for an initiative period of the programme. Among the diseases detectable by MS/MS, we prioritised which diseases are appropriate to be included in the study. We first held an expert panel of IEM specialists (DW, NV, PW, VS, SP, and CK) from four major hospitals in Bangkok where most of the IEM patients are treated to help with the selection of the diseases, including Siriraj Hospital, Ramathibodi Hospital, Chulalongkorn Hospital, and the Queen Sirikit National Institute of Child Health. The selection criteria were modified from the principle of population-based screening proposed by the World Health Organization (WHO) based on the recommendations of Wilson and Jungner [26] which included the magnitude of the health problem, availability of technology (screening and treatment), safety, and effectiveness of the treatment. As a result, six diseases consisting of PKU, isovaleric acidemia (IVA), methylmalonic acidemia (MMA), propionic acidemia (PA), maple syrup urine disease (MSUD), and multiple carboxylase deficiency (MCD) were selected for an economic evaluation.

### Study design

The cost-effectiveness analysis followed the standard guidelines of economic evaluations [27, 28] with present health technology services as the comparator. Thus, the analysis compared: 1) the current practice—or “pre-expanded newborn screening programme”—where only PKU is screened using the Guthrie test and PKU patients received early treatment whereas the other diseases detected were treated after symptomatic presentation; and 2) the “expanded newborn screening programme using MS/MS” where the six prioritised diseases were screened and treatment was given early or before symptomatic presentation. The costs and health outcomes of these alternatives were then compared by taking the societal perspective into account as suggested by Thai Health Technology Assessment guidelines [29].

The health outcomes of interest were measured in life-year gained (LY) and quality-adjusted life year (QALY) gained. A discount rate of 3% was applied for both the cost and outcome [30]. All costs were subsequently converted to year 2013 adjusted using the consumer price index medical care for medical goods and services and general consumer price index for those non-medical and other costs as recommended in the Thai health technology assessment guidelines [31]. The analyses were performed in Microsoft Excel 2007 (Microsoft Corp., Redmond, WA) and the results were presented as an incremental cost-effectiveness ratio (ICER) in Thai baht (THB) per QALY gained. For intercountry comparisons, costs can be converted into international dollars (I$) using the purchasing power parity (PPP) exchange rate of 1 I$ = 17.79 THB (2013) [32]. This analysis used the cost-effectiveness ceiling threshold of one times the gross domestic product (GDP) per capita (120,000 THB ≈ 6,745 I$) per QALY gained as recommended by the Health Economic Working Group under the Subcommittee for Development of the National List of Essential Drugs and the Subcommittee for Development of the Health Benefit Package and Service Delivery of the NHSO, Thailand [33].

### Analytical model

**Fig 1:**
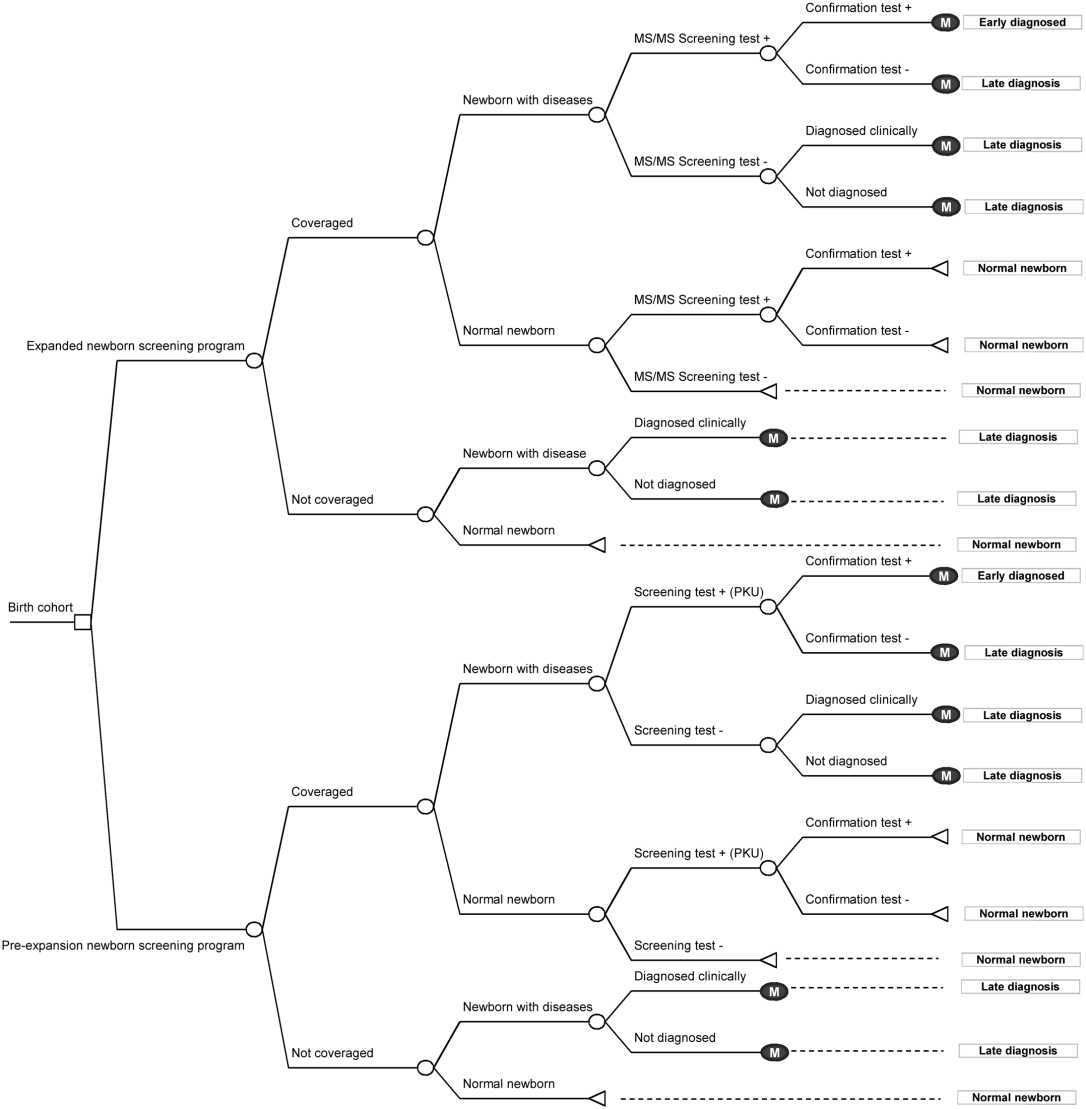
Simplified-decision-tree. Comparing the strategies of expanding the newborn screening programme with the pre-expanded newborn screening programme. MS/MS = Tandem mass spectrometry; PKU = phenylketonuria; M = Markov model.

A probabilistic multivariate model was conducted using a combination of a decision-tree and Markov model that followed a cohort of newborns with a cycle-length of one year. Our model consisted of newborns at birth starting either at the stage of being at risk for one of the six selected diseases or the normal newborn stage. Next, these newborns were able to transit through three possible scenarios consisting of early diagnosis, late diagnosis, and normal newborns (Fig 1). After that, the affected newborns were followed by applying a Markov model to capture possible changes in the health status for each year of life within the designated cycle-length for 100 years or lifetime. This is to ensure that all cost and outcomes related to the disease and intervention were comprehensively accounted for.

**Fig 2:**
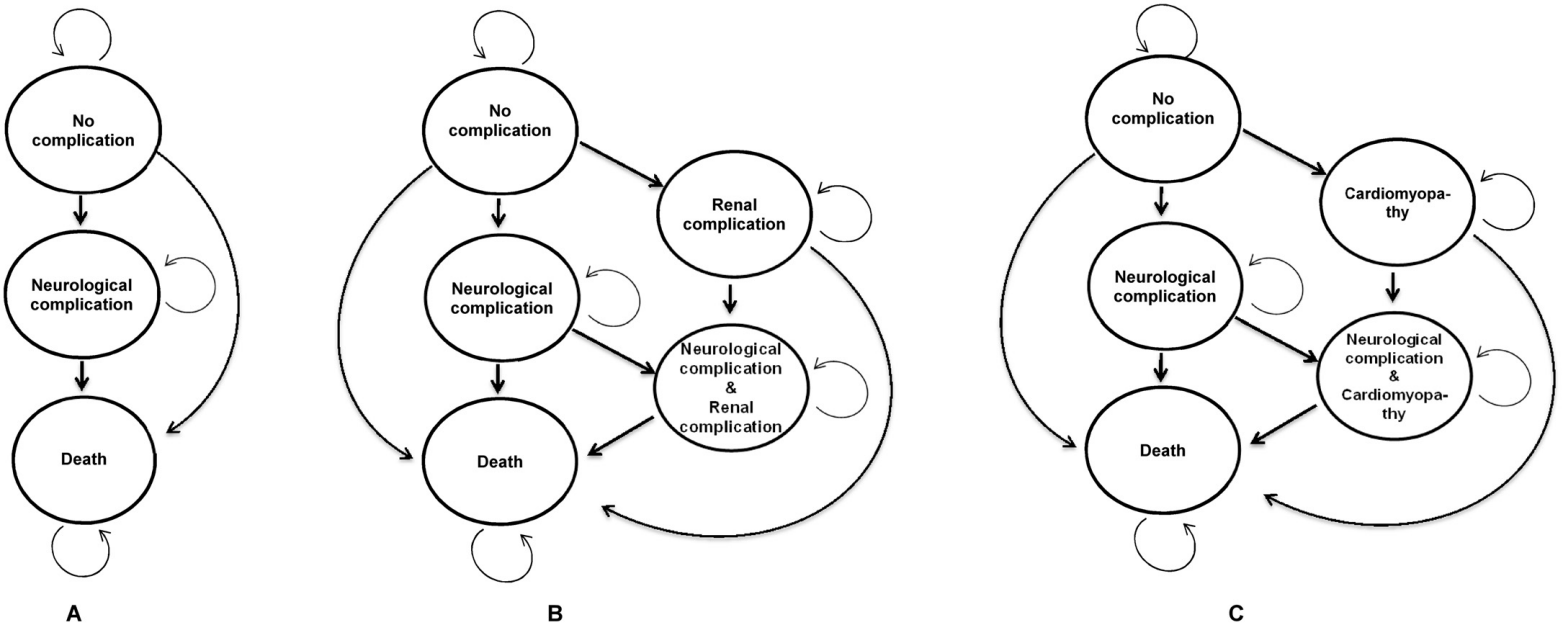
Markov model. Health states transition of selected IEM. (A) represents PKU, IVA, MSUD and MCD; (B) represents MMA; and (C) represents PA. PKU = phenylketonuria; IVA = isovaleric acidemia; MMA = methylmalonic acidemia; PA = propionic acidemia; MSUD = maple syrup urine disease; MCD = multiple carboxylase deficiency.

Although the six prioritised IEM diseases have the potential to cause several severe clinical manifestations, only the most common long-term complications were taken into account as health states in this analysis (Fig 2). Since it was highly possible that a majority of IEM patients would have neurological complications, this complication was deemed integral to the model [1]. The other important long-term complications represented in the model were renal failure in MMA and cardiomyopathy in PA [34]. Thus, the health states applied in each disease were divided into three different groups based on the most common long-term complications: 1) PKU, IVA, MSUD, and MCD (Fig 2A) were designated the health states of living without any complications, having neurological complications, and death; 2) MMA (Fig 2B) was consisted of the health states of living without any complications, having neurological complications, having renal failures, having both neurological and renal complications, and death; and 3) PA (Fig 2C) was presented the health states of living without any complications, having neurological complications, having cardiomyopathy, having both neurological complications and cardiomyopathy, and death.

### Estimation of disease incidences

The incidence of PKU in the Thai population is 2.22 per 100,000 live births. This data was obtained through a newborn screening programme at Siriraj Hospital—which had screened over 180,000 infants born in Bangkok Metropolitan—as well as a continuity programme for screening-positive cases [35]. Due to the lack of existing data on the incidences of the other IEM in Thai setting, the present study adopted the incidences of other Asian populations—specifically the Chinese and Japanese—which were considered comparable to Thai ethnicity. The incidence of the remaining five IEM ranged from 0.54 to 2.69 cases per 100,000 live births [7] [8] (Table 1).

**Table 1:** Mean and standard error (SE) of transitional probabilities used in the model.

Parameter		Distribution	Mean	SE	Reference
Uptake rate		Beta	0.97	0.0001	[38]
MS/MS sensitivity		Beta	1.00	-	[19]
MS/MS specificity		Beta	1.00	-	[19]
Guthrie sensitivity		Beta	0.9850	0.01	[39]
Guthrie specificity		Beta	0.9995	0.01	[39]
**Incidence of diseases**					
PKU		Beta	2.22ˣ10^−5^	1.11ˣ10^−5^	[35]
IVA		Beta	1.08ˣ10^−5^	5.38ˣ10^−6^	[8]
MMA		Beta	2.69ˣ10^−5^	8.50ˣ10^−6^	[8]
PA		Beta	5.40ˣ10^−6^	3.80ˣ10^−6^	[8]
MSUD		Beta	1.08ˣ10^−5^	5.38ˣ10^−6^	[8]
MCD		Beta	6.60ˣ10^−6^	3.30ˣ10^−6^	[7]
**Death from the disease** ^a^					
Yearly probability					
PKU	Age 0 to < 10 years	Beta	2.53ˣ10^−3^	-	Estimated from [36]
	Age 10 to < 20 years	Beta	2.88ˣ10^−2^	-	
	Age 20 to < 30 years	Beta	4.57ˣ10^−3^	-	
	Age 30 to < 40 years	Beta	4.39ˣ10^−3^	-	
	Age 40 to < 50 years	Beta	3.54ˣ10^−3^	-	
	Age 50 to < 60 years	Beta	3.24ˣ10–3	-	
	Age ≥ 60	Beta	1.13ˣ10–1	-	
Survival analysis					
IVA	Constant for baseline hazard	Lognormal	-2.515	0.721	Medical record review
	Ancillary parameter in Weibull distribution	Lognormal	-1.424	0.371	Medical record review
MMA	Constant for baseline hazard	Lognormal	-4.070	1.690	Medical record review
	Ancillary parameter in Weibull distribution	Lognormal	-0.865	0.532	Medical record review
PA	Constant for baseline hazard	Lognormal	-3.970	1.662	Medical record review
	Ancillary parameter in Weibull distribution	Lognormal	-0.745	0.477	Medical record review
MSUD	Constant for baseline hazard	Lognormal	-4.790	1.123	Medical record review
	Ancillary parameter in Weibull distribution	Lognormal	-0.665	0.289	Medical record review
**Long-term complications** (Yearly probability)					
PKU	Neurological complication	Beta	0.1340	0.0852	[37]
IVA	Neurological complication	Beta	0.0509	0.0549	Medical record review
MMA	Neurological complication	Beta	0.0897	0.0730	Medical record review
	Renal failure	Beta	0.0339	0.0487	Medical record review
PA	Neurological complication	Beta	0.6838	0.2080	Medical record review
	Cardiomyopathy	Beta	0.0468	0.1056	Medical record review
MSUD	Neurological complication (Age 0 to <1 year)	Beta	0.2778	0.1056	Medical record review
	Neurological complication (Age 1 to < 2 years)	Beta	0.3846	0.1147	Medical record review
	Neurological complication (Age = >2)	Beta	0.6250	0.1122	Medical record review
MCD	Neurological complication (Age 0 to < 3 years)	Beta	0.0572	0.0774	Medical record review
	Neurological complication (Age 3 to < 7 years)	Beta	0.0646	0.0819	Medical record review
	Neurological complication (Age = > 7 years)	Beta	0.0218	0.0487	Medical record review
**Relative risk of early compared with clinical diagnosis**					
PKU	Mortality	Beta	0.67	-	[36]
	Neurological complication (RR)	Lognormal	0.02	0.03	[37, 40, 41]
IVA	Mortality reduction	Beta	0.20	-	[42]
	Neurological complication (RR)	Lognormal	0.28	0.11	[43]
MMA	Mortality reduction	Beta	0.25	-	[42]
	Neurological complication (RR)	Lognormal	0.63	0.41	[34]
	Renal failure (RR)	Lognormal	0.33	0.31	[34]
PA	Mortality reduction	Beta	0.25	-	[42]
	Neurological complication (RR)	Lognormal	0.73	0.30	[34]
	Cardiomyopathy (RR)	Lognormal	0.46	0.95	[44]
MSUD	Mortality reduction	Beta	0.20	-	[42]
	Neurological complication (RR)	Lognormal	0.23	0.11	[45, 46]
MCD	Mortality reduction	Beta	1.00	-	[47]
	Neurological complication (RR)	Lognormal	0.00	-	[14, 47]

^a^ See S1 Table for death from other causes.

PKU = phenylketonuria; IVA = isovaleric acidemia; MMA = methylmalonic acidemia; PA = propionic acidemia; MSUD = maple syrup urine disease; MCD = multiple carboxylase deficiency; RR = Relative risk of early-diagnosed patients compared with clinical diagnosed patients

### Transitional probabilities of long-term complications and mortality

Transitional probabilities, or *tp(u)* (i.e., transition to long-term complications and/or death), are required for the Markov model to simulate the events of patients entering each health state (Table 1). Our base-case analysis mainly estimated a baseline rate of long-term complications using a retrospective review of the registered medical records of patients with the six IEM diseases at the four hospitals from 1992 to 2012. The data consisted of clinical variables such as the survival time, demographics, clinical complications, and patient status (alive/dead) of 119 patients (IVA = 23, MMA = 20, PA = 8, PKU = 26, MSUD = 32, and MCD = 10). The annual transitional probabilities of long-term complications were estimated by employing nonparametric methods and the annual probabilities of mortality were estimated using survival analysis.

A parametric survival-time model was applied in order to derive a time-dependent probability of mortality for IVA, MMA, PA, and MSUD. In particular, a Weibull model which was compared with exponential model was used as the AIC (Akaike information criterion) indicated that it was more fit to the actual data. The survival function, *S(t)* which describes the probability of survival as a function of time is [48]:

S(t)=exp{−H(t)} (1)

and

$$H(t)=\lambda t^{\gamma }$$ (2)

where *H(t)* which is the cumulative hazard; λ (lambda) is the scale parameter; *t* is time in years; and and ancillary or γ (gamma) is the shape parameter that describes the instantaneous death rate, the hazard rate *h(t)*, which increase which time if γ > 1.

The transitional probability of dying during the cycle, *tp(u)*, is therefore estimated from the following formula (where *u* is the cycle-length of the model):

$$tp(u)=1-\text{exp}\left\{\lambda {\left(t-u\right)}^{\gamma }-\lambda t^{\gamma }\right\}$$ (3)

For PKU and MCD, the reviewed data showed that patients who were admitted to hospitals were all still alive which prevented us from capturing their lifespan by applying survival analysis. In the case of PKU, the probability was adopted from the data of the United States’ national survey on PKU[36] whereas the lack of long-term literature on MCD patients required the experts to make the assumption that their mortality is equivalent to the normal population. In addition, for all six IEM, apart from the probability of mortality caused specifically by the diseases, the model also included death from other causes estimated from the Thai life table [49] (S1 Table).

### Sensitivity and specificity

At present, only PKU is screened using the Guthrie test. This traditional method yielded a sensitivity of 98.50% and specificity of 99.50% [39] as shown in Table 1.

Due to a prior systematic review supporting the high accuracy of MS/MS [19], we assumed a screening test sensitivity of 100% and specificity of 100% (Table 1). The expert panel also agreed with this assumption that a sensitivity and specificity of 100% for both should be used. As such, this meant that no IEM cases were missed by the MS/MS screening programme.

### Outcome of early detection

A relative risk (RR) was used as the main outcome measure. The RR of the experimental or screened group compared with the clinical diagnosed group on neurological complications or other complications of each IEM disease—was obtained based on a systematic search through Ovid MEDLINE and Embase. Detailed information about the systematic search is available in (S1 Search Strategy). Where data was available, pooling RR was performed (Table 1). The effectiveness in the reduction of neurological complication of patients with early detection of PKU was calculated based on data from three cohort studies consisting of: a collaborative study of children treated for PKU in the US [41]; a study in Italy from the Regional Center for Inborn Errors of Metabolism [40]; and a retrospective study of PKU patients diagnosed and controlled in Spain [37] (RR 0.02, 95%CI (0.00–0.09)). For IVA patients, the reduction in risk of neurological complications was estimated based on a literature review investigating 155 IVA patients worldwide (RR 0.28, 95%CI (0.07–0.50)) [43]. Two case series reports of MSUD patients diagnosed in the US [46] and Japan [45] were pooled to estimate the reduction of neurological complications (RR 0.23, 95%CI (0.01–0.45)). For MMA and PA patients, the review study of cases around the world comparing symptomatic cases and those diagnosed through newborn screening [34] was used to calculate the RR of neurological complications (RR 0.63, 95%CI (0.00–1.00) for MMA, and RR 0.73, 95%CI (0.00–1.00) for PA) as well as the RR of renal failure in MMA patients (RR 0.33, 95%CI (0.00–0.93)). The RR of cardiomyopathy in PA patients was 0.46 (95%CI (0.00–1.00)) based on a German study comparing PA patients diagnosed through newborn screening and those obtained through clinical diagnosis [44]. In the case of MCD, we assumed no long-term complications if early detection and treatment was provided as results according to the report of American Academy of Paediatrics [47] and previously published data [14] showed.

An extensive search of literature was also conducted to determine the benefit of early detection for reduction of mortality in patients but no any robust evidence-based information was found. Nevertheless, the potential data found could be an estimation of UK paediatrician on reduction on death of early-diagnosed IVA, MMA, PA, and MSUD patients ranged between 20%-25% given to a previous study [42]. So, the information was adopted into the model. For PKU and MCD patients, normal life expectancy was assumed for the effectiveness of early detection which was supported by the data from medical record reviews which showing no PKU and MCD patients died due to the disease (Table 1).

### Screening costs

Two sample quotations of MS/MS manufacturers and distributors were obtained to estimate the capital and material costs of the MS/MS screening programme. In terms of human resources, a proportion of labour costs to capital and material costs from international publications [15, 16, 19] were estimated and applied to the analysis (Table 2).

**Table 2:** Means and standard error (SE) of cost parameters presented in 2013 Thai Baht. 1 I$ = 17.79 THB.

Parameter		Distribution	Mean	SE	Reference
Screening cost for MS/MS (per sample)		Gamma	294	126	Survey
Screening cost for Guthrie (per sample)		Gamma	5.00	-	NSCO
Confirmation cost (per sample)		Gamma	2,349	168	Hospital database
Hospital inpatient care (IPD) cost per year					
First year of treatment					
PKU		Gamma	80,316	19,899	Hospital database
IVA		Gamma	174,006	53,349	Hospital database
MMA		Gamma	252,457	81,466	Hospital database
PA		Gamma	284,718	91,965	Hospital database
MSUD		Gamma	252,859	53,281	Hospital database
MCD		Gamma	92,070	25,721	Hospital database
Second year of treatment onward					
IVA		Gamma	21,290	3,712	Hospital database
MMA		Gamma	191,729	73,046	Hospital database
PA		Gamma	160,951	44,432	Hospital database
MSUD		Gamma	52,580	15,029	Hospital database
MCD		Gamma	69,615	60,111	Hospital database
Hospital outpatient care (OPD) cost per year					
PKU		Gamma	16,366	321	Hospital database
IVA		Gamma	44,925	1,335	Hospital database
MMA		Gamma	108,671	2,272	Hospital database
PA		Gamma	61,328	2,142	Hospital database
MSUD		Gamma	17.928	519	Hospital database
MCD		Gamma	4,055	362	Hospital database
Pharmaceutical product per year^a^					
L-carnitine (IVA, MMA)		Gamma	1,470	-	Survey
L-glycine (IVA)		Gamma	276	-	Survey
Cobalamin (MMA, PA)		Gamma	7,350	-	Survey
Biotin (PA)		Gamma	13,597	-	Survey
Biotin for (MCD)		Gamma	10,198	-	Survey
Metabolic formula per year					
PKU	Age 0 to < 4 years	Gamma	75,511–81,552	-	Survey
	Age ≥4 years	Gamma	45,306	-	Survey
IVA	Age 0 to < 5 years	Gamma	51,347–78531	-	Survey
	Age ≥5 years	Gamma	45,306		Survey
MMA	Age 0 to < 7 years	Gamma	51,347–75,511	-	Survey
	Age ≥7 years	Gamma	45,306		Survey
PA	Age 0 to < 7 years	Gamma	51,347–75,511	-	Survey
	Age ≥7 years	Gamma	45,306		Survey
MSUD	Age 0 to < 4 years	Gamma	55,878–86,082	-	Survey
	Age ≥4 years	Gamma	45,306		Survey
Direct non-medical cost per year					
PKU	with long-term complications	Gamma	27,704	-	Survey
	without long-term complications	Gamma	12,941	9,768	Survey
IVA	with long-term complications	Gamma	27,704	-	Survey
	without long-term complications	Gamma	15,781	4,924	Survey
MMA	with long-term complications	Gamma	46,516	20,107	Survey
	without long-term complications	Gamma	22,408	15,429	Survey
PA	with long-term complications	Gamma	36,348	26,618	Survey
	without long-term complications	Gamma	22,408	15,429	Survey
MSUD	with long-term complications	Gamma	45,770	22,843	Survey
	without long-term complications	Gamma	22,408	15,429	Survey
MCD	with long-term complications	Gamma	27,704	-	Survey
	without long-term complications	Gamma	14,361	7,346	Survey
Productivity cost per year					
PKU	with long-term complications	Gamma	26,522	2,235	Survey
	without long-term complications	Gamma	127,896	-	Survey
IVA	with long-term complications	Gamma	129,930	-	Survey
	without long-term complications	Gamma	43,944	5,469	Survey
MMA	with long-term complications	Gamma	177,404	20,107	Survey
	without long-term complications	Gamma	50,880	11,493	Survey
PA	with long-term complications	Gamma	128,784	11,010	Survey
	without long-term complications	Gamma	50,880	11,493	Survey
MSUD	with long-term complications	Gamma	105,717	9,573	Survey
	without long-term complications	Gamma	50,880	11,493	Survey
MCD	with long-term complications	Gamma	129,930	-	Survey
	without long-term complications	Gamma	35,444	85,000	Survey

^a^Calculated at patient weight 1 kilogram.

NSCO = Neonatal Screening Operation Centre; PKU = phenylketonuria; IVA = isovaleric acidemia; MMA = methylmalonic acidemia; PA = propionic acidemia; MSUD = maple syrup urine disease; MCD = multiple carboxylase deficiency

To screen around 750,000 births per year, we estimated that Thailand requires 7 to 9 MS/MS machines. When requesting for the sample quotations, we received a difference in terms of price. The cost per MS/MS machine ranged from 9.8 to 15 million THB in addition to an annual maintenance fee of 1.8 to 4.8 million THB. To calculate for depreciation, the equipment would have a lifetime of 7–8 years life without the salvage value. The prices of reagent per sample were also considerably dissimilar at 111 and 300 THB per sample. Meanwhile, the labour cost accounted for approximately 28% of the screening cost and was calculated to be 64 THB per sample. Thus, from this information, the cost of MS/MS screening per sample was estimated to be 294 THB per sample (Table 2).

### Treatment costs

This study was approached from a societal perspective so all costs relevant to patient, health care system, and society were analysed. A cost analysis was conducted specifically for each of the six prioritised diseases. Treatment costs mainly included hospitalisation expenses and dietary management. Retrospective information of the treatment costs for the IEM patients was collected from each of the four hospitals’ databases, and then the resulting data were pooled and analysed together (Table 2).

From the data, it was evident that the cost of inpatient care (IPD) was very high in the first year of treatment due to an acute phase. The treatment cost decreased dramatically in the second year of treatment. Thus, the IPD cost was divided into two periods: 1) the first year of treatment; and 2) the second year of treatment onwards. Regarding the cost of outpatient care (OPD), the cost per year was estimated by multiplying the cost per visit by the number of outpatient visits per year (S2 Table).

Additionally, the cost of the supplemental metabolic formula was calculated based on the assumption that patients needed a special formula for their lifespan to maintain normal metabolic function. Price of this special product was provided by manufacturer and the data of quantity used for patients was obtained from the IEM specialists. Regarding the cost of other supplemental products such as orphan drugs, vitamins, and cofactors, the IEM specialists were asked to answer a set of questions on a provided questionnaire survey about the type, the quantity, and the unit price of the product being used to treat their IEM patients. For productivity loss and direct-non medical costs, data were collected via face-to-face interviews with the patients’ families using a structured questionnaire. The parents of the IEM patients were asked about the time spent to look after their children who have been disabled due to complications caused by IEM. The average wage in Thailand [50]—classified by gender and age—was used for calculating the productivity loss or opportunity cost. We also inquired about costs relevant to hospitalisation such as travel costs from home to the hospital. From the data obtained, the differences between the cost of mild and severe cases were observed, and the costs for these cases were therefore classified into two groups according to the severity/complication of the disorder(s), including without complications and with complications (Table 2).

### Utility measurement

There are several concerns about assessing health utility weight from children, especially an available and appropriate measuring instrument allowing children to complete their health status [51]. In addition, both small number of living patients and their intellectual disabilities status are factors that make it possibly impossible to perform primary data collection. Therefore, the estimation of health utility was conducted by holding an expert panel consisting of the six IEM specialists as proxies.

EuroQoL Five-Dimension Questionnaire (EQ-5D) was applied as a tool to estimate health utility weight of each health stage of each disease. The IEM specialists were then asked to recall from their current as well as previous IEM patients. Then, they filled out a score onto the prepared paper sheet. Subsequently, the average score was presented and discussed among the experts. As a result, a consensus for health utility weight was reached and applied into the model (Table 3).

**Table 3:** Health utility weight of IEM patients.

Utility estimated		Distribution	Mean	SE	Reference
PKU	without long-term complication	Beta	0.71	0.02	Expert panel
	with mental retardation	Beta	0.13	0.19	Expert panel
IVA	without long-term complication	Beta	0.71	0.07	Expert panel
	with mental retardation	Beta	0.00	0.15	Expert panel
MMA	without long-term complication	Beta	0.62	0.06	Expert panel
	with mental retardation	Beta	0.16	0.18	Expert panel
	with renal failure	Beta	0.45	0.16	Expert panel
	with mental retardation and renal complication	Beta	0.14	0.22	Expert panel
PA	without long-term complication	Beta	0.49	0.13	Expert panel
	with mental retardation	Beta	0.05	0.21	Expert panel
	with cardiomyopathy	Beta	0.41	0.20	Expert panel
	with mental retardation and cardiomyopathy	Beta	0.00	0.28	Expert panel
MSUD	without long-term complication	Beta	0.60	0.07	Expert panel
	with mental retardation	Beta	0.00	0.04	Expert panel
MCD	without long-term complication	Beta	0.84	0.11	Expert panel
	with mental retardation	Beta	0.51	0.07	Expert panel

PKU = phenylketonuria; IVA = isovaleric acidemia; MMA = methylmalonic acidemia; PA = propionic acidemia; MSUD = maple syrup urine disease; MCD = multiple carboxylase deficiency

### Uncertainty analysis

To investigate the robustness of the cost-effectiveness results, we performed two types of uncertainty analysis. The first was a one-way sensitivity analysis which examined the effect of changes in key parameters on the ICERs of the base-case scenario. These variables included: the incidence of six selected IEM; probability of long-term complications; the effectiveness of the screening intervention (RR); health utility weight of six selected IEM; first year IPD and OPD costs; second year direct medical costs; metabolic formula and pharmaceutical product costs; direct non-medical cost of patients with complication; direct non-medical cost of patients without complication; productivity loss of patients with complication; productivity loss of patients without complication; uptake rate of screening; and MS/MS screening cost. Since each of these variables (except for the uptake rate and the screening cost) comprised six different values which varied by disease, we assumed that these values simultaneously changed in the same direction to the lower or upper bound once each variable was examined. The value being tested varied based on a 95% confidence interval (CI) of these parameters with standard errors. The costs of the metabolic formula and pharmaceutical products—which are parameters without standard error—were assumed to be varied by 50% from their mean value. Other parameters (not one being tested) were randomly generated by using a probabilistic sensitivity method.

The second, a probabilistic sensitivity analysis (PSA), was also conducted to assess the uncertainty involving all model parameters according to their mean, standard error (SE), and distribution shown in Tables 1 to 3. Probability distributions were defined as follows: (1) beta-distributions were assigned where parameter values ranged from zero to one, such as transition probabilities and utility parameters; (2) gamma-distributions were specified when parameter values were above zero and positively skewed by costs variables; and (3) a log-normal distribution was used for survival parameters and RR. A Monte Carlo simulation performed in Microsoft Excel 2007 (Microsoft Corp., Redmond, WA) was employed to generate 1,000 rounds of the simulation to demonstrate a range of plausible lifetime costs, health outcomes (LYs and QALYs), and ICERs. The result of the analysis was plotted in a cost-effectiveness plane. Moreover, the result was further analysed for a relationship between the values of the ceiling ratio and the likelihood of favouring each screening strategy as the result is illustrated using cost-effectiveness acceptability curves showing.

### Budget impact analysis

Based on the model, a budget impact analysis (BIA) was also conducted by following the standard BIA frameworks for healthcare intervention in Thailand [52] along with international protocol [53]. The analysis applied the perspective of the budget holder in Thailand, i.e. the NHSO, and aimed to project the financial plans between the implementation of the new screening programme and the status quo. The costs were analysed and reported into two categories: screening cost and treatment cost. The costs were inflated at 0.5% each year [31] with a time horizon of 10 years. Since there is a stable trend for population growth in Thailand, the annual cohort of newborns was fixed at 750,000 per year [54]. The analysis was based on the important assumption that the new programme will be managed by the existing screening organization, i.e. the neonatal screening operation centre, and will replace the current PKU screening. Therefore, there is no cost of setting up a new department in order to handle the programme. The machine cost was considered as a fixed capital cost and was spread out equally throughout each year of the programme based on the concept of equivalent annual cost [55], while reagent and administrative costs were variable costs dependent on the number of participants. We conservatively assumed that the uptake for the new screening programme was 80% for the first year of the implementation and then increased it to 85%, 90%, 95%, and 100% in subsequent years. The minimum cost and maximum cost scenarios of the screening budget was examined based on the range of 95% CI of screening costs, while the scenario of the treatment budget was tested based on the incidence of disease (varying the incidence of all diseases simultaneously to the lower and upper bounds).

## Results

### Cost-effectiveness analysis of the pre-expanded and the expanded newborn screening programme

The cost-effectiveness analysis via the adoption of a new screening strategy compared with the existing screening programme indicate ICERs of 602,606 THB per LY gained (33,873 I$ per LY gained) and 1,043,331 THB per QALY gained (58,647 I$ per QALY gained) (Table 4), both of which are above the agreed threshold currently used in Thailand.

**Table 4:** Costs, health outcomes, and incremental cost-effectiveness ratios (ICERs) of two neonatal screening programmes. 1 I$ = 17.79 THB. THB = Thai baht; LYs = life-years; ICER = incremental cost effectiveness ratio; QALYs = quality-adjusted life year.

Strategy	Total cost (THB)	Incremental Cost (THB)	LYs	LY gained	ICER (THB/LY gained)	QALYs	QALY gained	ICER (THB/QALY gained)
Pre-expanded newborn screening programme	153.27	—	66.42256	—	—	66.42229	—	—
Expanded newborn screening programme	676.55	523.28	66.42343	0.00087	602,606	66.42279	0.00050	1,043,331

In order to understand the potential benefit of screening at the individual diseases level, Table 5 illustrates the lifetime outcome of each affected child once they are detected early or late. The analysis suggests that early medical treatment substantially improves the health outcome in PKU patients resulting in 9.60 QALY higher than those with late detection. IVA patients also noticeably benefit from early detection which extends their QALY by 3.69. In MSUD patients, early detection also yields better health outcomes that help prolong QALY by 2.73. In terms of lifetime costs, the difference between providing early healthcare and late healthcare to those with MSUD, IVA, and PKU ranges between 200,045 THB (11,245 I$) and 502,913 THB (28,269 I$) per patient. MCD patients gain 1.66 QALY from early detection and it is the only disease where early medical management reduces the patient’s lifetime costs (-256,779 THB (-14,434 I$)). However, in most diseases, the costs of giving lifetime care in patients detected early are higher than those in clinically diagnosed patients; it is clear that being detected early requires significantly much more costs in MMA and PA patients (1,783,826 THB (100,271 I$) and 1,241,945 THB (69,811 I$), respectively), which is obviously high relative to their health outcome gained (2.83 QALY and 0.81 QALY for MMA and PA, respectively).

**Table 5:** Difference of lifetime health outcomes and costs per patient after early detection or late detection. 1 I$ = 17.79 THB. The result was under adjusting of 3.0% discounting rate. Undiscounted version was provided as (S3 Table). PKU = phenylketonuria; IVA = isovaleric acidemia; MMA = methylmalonic acidemia; PA = propionic acidemia; MSUD = maple syrup urine disease; MCD = multiple carboxylase deficiency; RR = Relative risk of early-diagnosed patients compared with clinical diagnosed patients.

Disease	Cost (THB)			Life-years			QALYs		
	Early diagnosis	Late diagnosis	Difference	Early diagnosis	Late diagnosis	Difference	Early diagnosis	Late diagnosis	Difference
**PKU**	3,145,203	2,642,290	502,913	29.55	19.57	9.99	20.91	11.31	9.60
**IVA**	3,728,014	3,409,629	318,384	17.63	14.84	2.79	11.51	7.82	3.69
**MMA**	7,685,602	5,901,776	1,783,826	16.33	12.14	4.19	8.67	5.84	2.83
**PA**	3,838,684	2,596,739	1,241,945	8.82	5.70	3.12	1.25	0.74	0.81
**MSUD**	3,462,620	3,262,575	200,045	14.88	12.64	2.24	3.93	1.20	2.73
**MCD**	2,544,647	2,801,427	-256,779	29.59	28.77	0.81	24.75	23.09	1.66

### Uncertainty analysis

**Fig 3:**
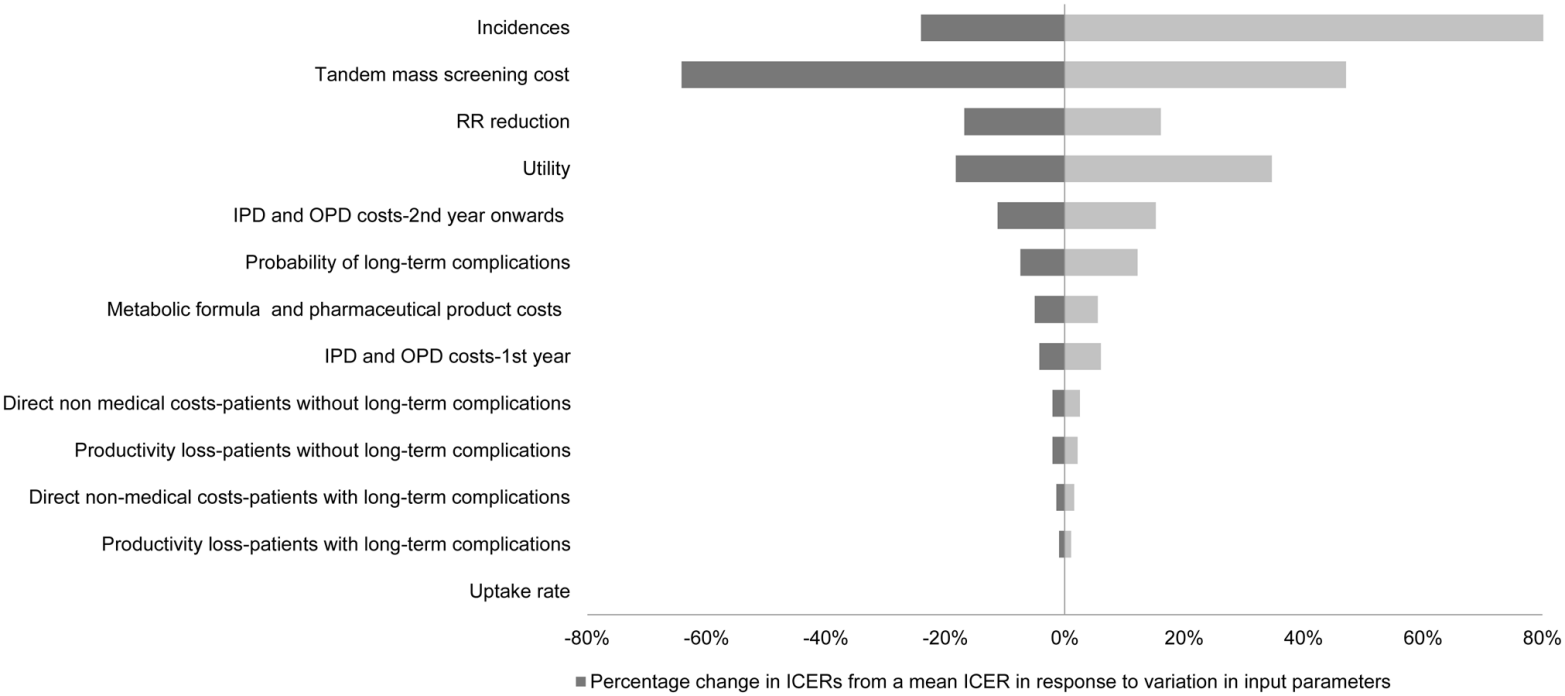
One-way sensitivity analysis. Tornado graph showing results of one-way sensitivity analysis derived from probabilistic method. These figures indicating parameters which have the largest effect on incremental cost effectiveness ratio or ICER (THB per QALY gained) when they are varied individually. IPD = cost of inpatient care; OPD = cost of outpatient care.

The results of the one-way sensitivity analysis using probabilistic model was further elaborated upon using a tornado diagram as shown in Fig 3, indicating that the most sensitive factor to ICER (THB per QALY gained) was the incidence. This was followed by: the MS/MS screening cost; RR reduction; and health utility. Among the factors least sensitive to the results were the uptake rate, productivity loss, and direct non-medical costs.

**Fig 4:**
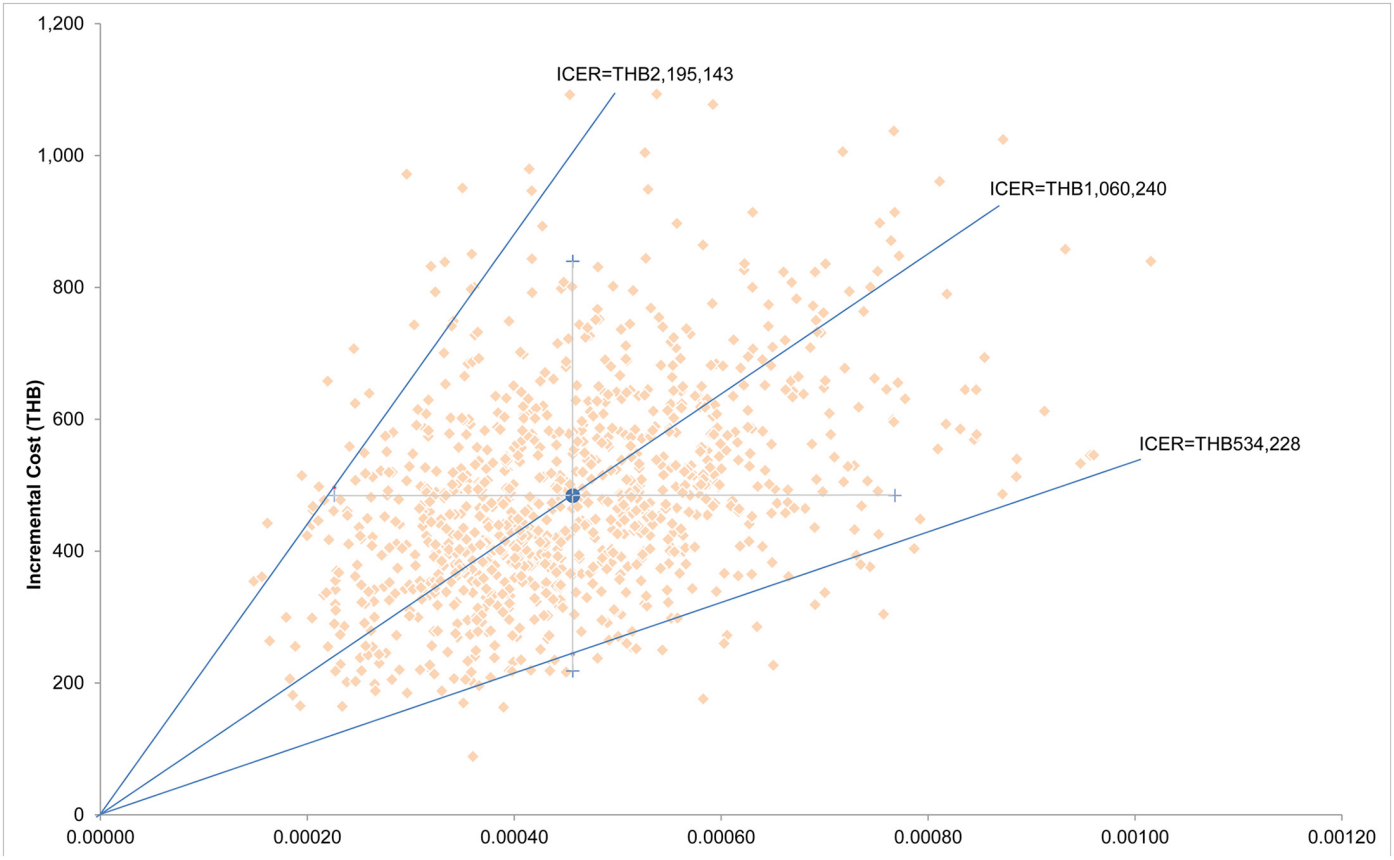
Cost-effectiveness plane. Monte Carlo simulation results on cost-effectiveness plane for the expanded newborn screening showing interval estimates for cost, outcome and incremental cost-effectiveness ratio. The figure shows the horizontal I-bar representing the 95% uncertainty interval on life-year gained, the vertical I-bar representing the 95% uncertainty interval on incremental cost, and the wedge represent the 95% uncertainty interval on the ICER. THB = Thai baht; ICER = incremental cost effectiveness ratio.

Fig 4 shows the result of the probabilistic sensitivity analysis. Monte Carlo simulation indicated that compared to the ‘current practice’ strategy, the ‘expanded newborn screening’ strategy was more costly but more effective in more than 95% of the simulated cases. The average patient with screening accrued 0.00048 (95% CI: 0.00023–0.00077), and THB 484.09 (95% CI: 218.25–839.59) more QALYs and costs than that without screening, giving an ICER of THB 1,060,240 (95% CI: 534,228–2,195,143) per QALY which, however, exceeds the threshold for cost-effective intervention in Thailand (THB 120,000 per QALY).

**Fig 5:**
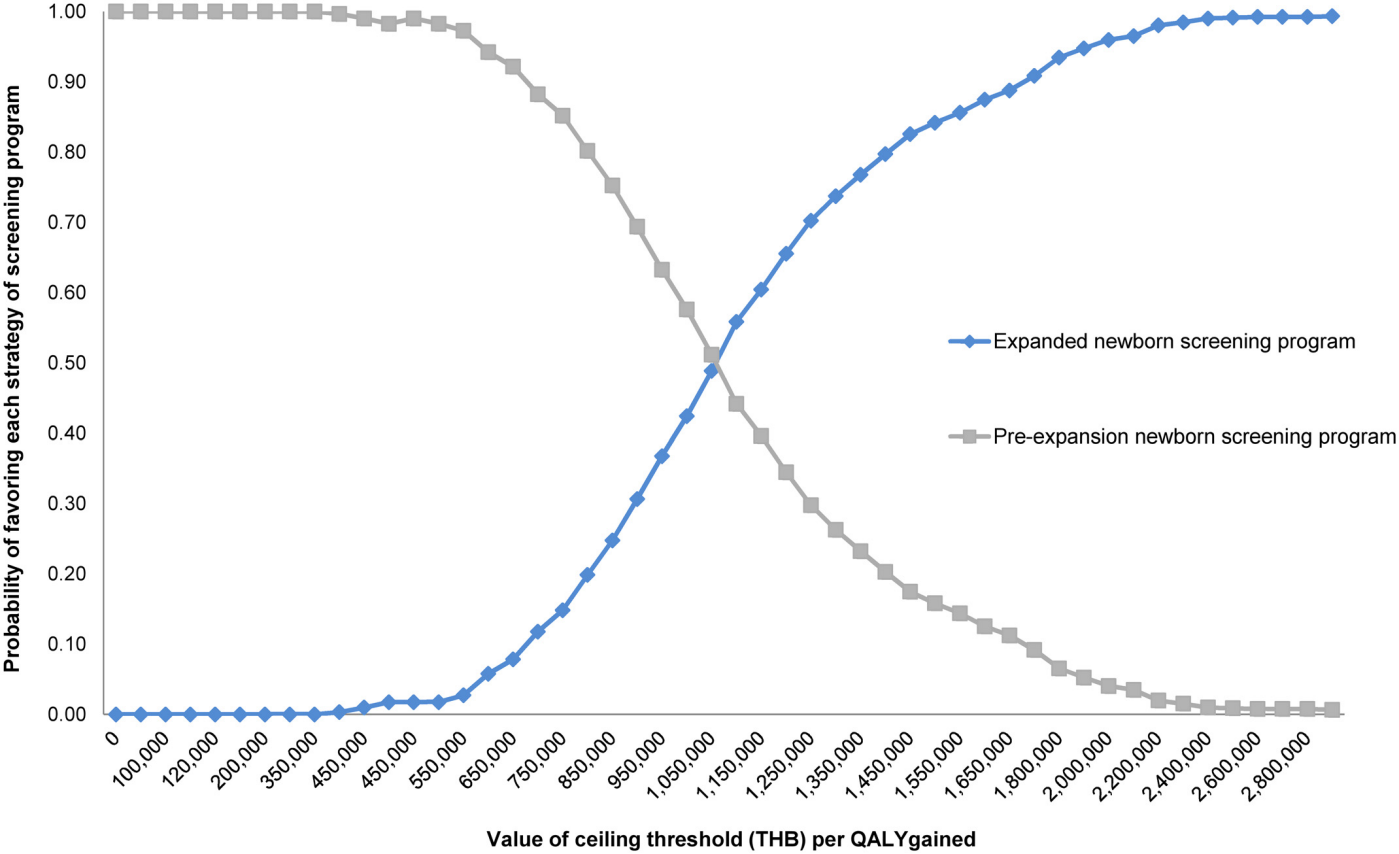
Acceptability curve. The graph shows the probabilities of each strategy being cost-effective at a given ceiling ratio. The dashed lines represent the willingness to pay thresholds for the adoption of health interventions in Thailand.

Fig 5 illustrates the cost-effectiveness acceptability curves representing the probability of both screening programme scenarios at different thresholds or willingness to pay being cost-effectiveness. When considering willingness to pay at less than Thailand’s value of one times the 2013 GDP per capita per QALY gained, the current screening programme has the potential to be more cost-effective. However, if the threshold is higher than approximately 1,100,000 THB per QALY gained, the MS/MS screening programme becomes a better option.

### Budget impact analysis

**Fig 6:**
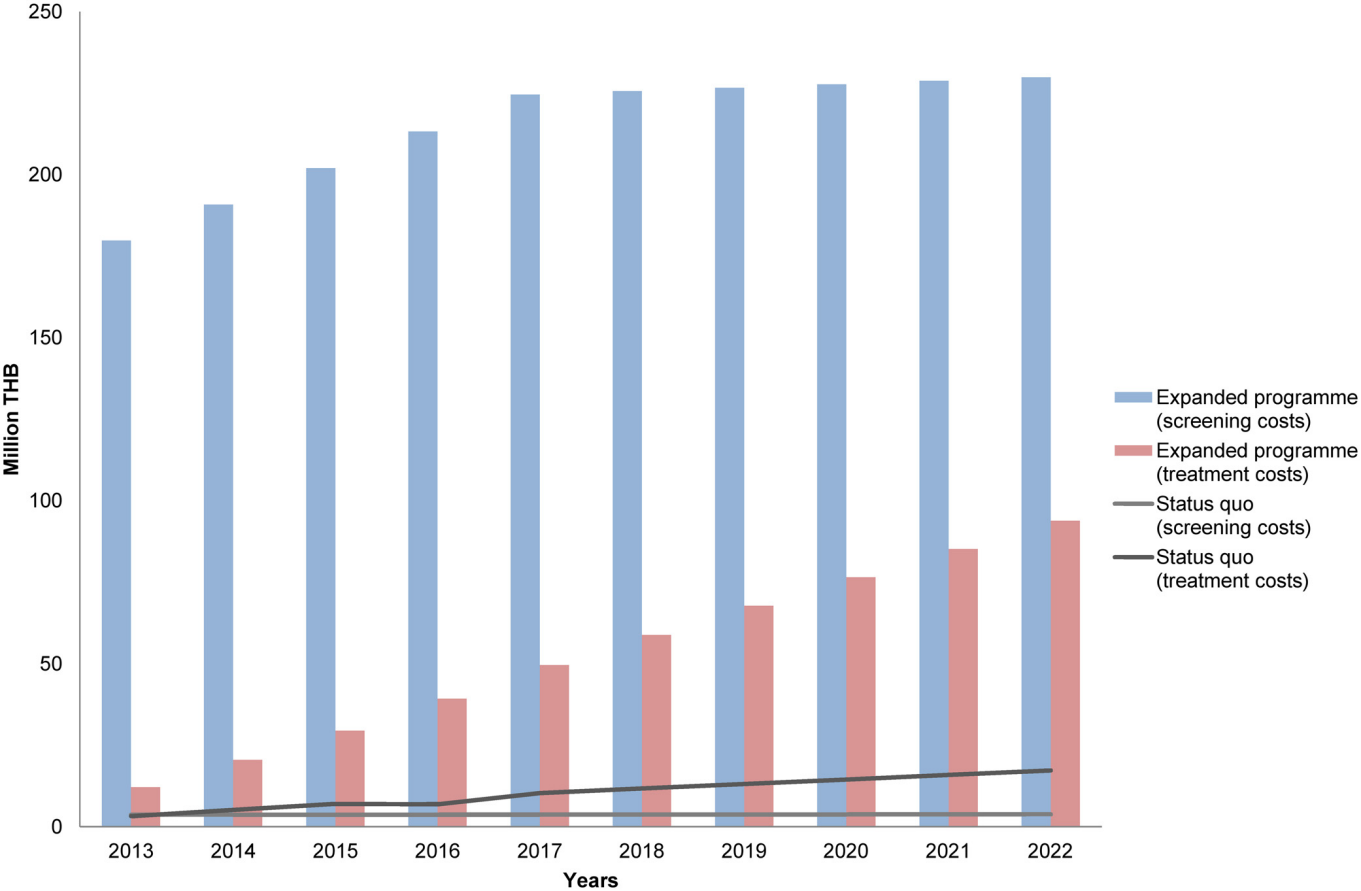
Budget Trend. The trend of the budget required for the screening programme compared with the current situation over ten years. THB = Thai baht.

From Table 6, it can be expected that the screening programme will have a total cost of 191.9 million THB per year in the earlier period and continually increase to above 300 million THB after seven years of implementation. In detail, the cost of screening is estimated to be 179.8 million THB in the first year and stays roughly at 200 million THB after three years of operation. Whereas the treatment cost is only a small proportion compared to the total cost in the earlier years (e.g. 12.1 million THB in 2013), it subsequently grows rapidly and is estimated to reach 93.9 million THB in the tenth year; this amount accounts for nearly one-third of the total cost in that year. It is obvious that the total expense of the screening programme is expected to increase overtime and the costs of treatment will be more and more substantial for the overall budget as illustrated in Fig 6. The analysis also found that there is a considerable switching cost from the current practice to the new programme, amounting to 2,539.6 million THB for the ten-year period.

**Table 6:** Estimated annual budget impact during 2013 to 2022 of the MS/MS screening programme implementation compared with the status quo (million THB). 1 I$ = 17.79 THB

Year^a^	Expanded newborn screening programme							Status quo							Difference
	Screening cost			Treatment cost			Total	Screening cost			Treatment cost			Total	Total
	Base case	Min	Max	Base case	Min	Max	(Base case)	Base case	Min	Max	Base case	Min	Max	(Base case)	(Base case)
2013	**179.8**	102.8	256.8	**12.1**	8.5	15.8	**191.9**	**3.6**	3.6	3.6	**3.2**	2.8	3.6	**6.8**	**185.0**
2014	**190.8**	109.4	272.3	**20.5**	14.4	26.7	**211.3**	**3.6**	3.6	3.6	**5.2**	4.4	6.0	**8.8**	**202.5**
2015	**202.0**	116.1	287.9	**29.4**	20.6	38.3	**231.4**	**3.7**	3.7	3.7	**7.1**	5.9	8.2	**10.7**	**220.7**
2016	**213.2**	122.9	303.6	**39.2**	27.5	51.0	**252.5**	**3.7**	3.7	3.7	**6.9**	7.4	10.5	**10.6**	**241.9**
2017	**224.6**	129.8	319.4	**49.6**	34.7	64.4	**274.1**	**3.7**	3.7	3.7	**10.3**	8.5	12.1	**14.0**	**260.1**
2018	**225.6**	130.8	320.4	**58.8**	41.2	76.5	**284.4**	**3.7**	3.7	3.7	**11.7**	9.7	13.8	**15.5**	**269.0**
2019	**226.7**	131.9	321.5	**67.7**	47.4	88.1	**294.4**	**3.7**	3.7	3.7	**13.1**	10.8	15.4	**16.9**	**277.5**
2020	**227.7**	132.9	322.5	**76.5**	53.6	99.5	**304.2**	**3.8**	3.8	3.8	**14.5**	11.9	17.1	**18.3**	**286.0**
2021	**228.8**	134.0	323.6	**85.2**	59.7	110.7	**314.0**	**3.8**	3.8	3.8	**15.9**	13.0	18.7	**19.7**	**294.3**
2022	**229.8**	135.0	324.6	**93.9**	65.7	122.0	**323.7**	**3.8**	3.8	3.8	**17.2**	14.2	20.3	**21.0**	**302.6**
Total	**2148.9**	1245.6	3052.3	**533.0**	373.3	692.8	**2681.9**	**37.1**	37.1	37.1	**105.2**	88.6	125.9	**142.4**	**2539.6**

^a^ The uptake rate was assumed at 80%, 85%, 90%, 95%, and 100% in 2013, 2014, 2015, 2016, and 2017–2022, respectively.

Min = minimum; Max = maximum

## Discussion

Based on the current threshold recommended in Thailand, the results of the present study suggest that implementing the MS/MS screening programme does not meet the criteria for cost-effectiveness. The incidence rate and the MS/MS screening cost are some of the major parameters influencing the cost-utility results followed by the RR reduction and health utility weight. A one-way sensitivity analysis shows that changing the parameters’ values did not affect the conclusion of the study. The result of the probabilistic analysis suggests that the better option is to continue implementing the Guthrie test for PKU. However, the benefits of early management for IVA, MSUD, PKU, and MCD patients are attractive. The budget impact analysis suggests that the likely costs of implementing the programme is about 2,700 million THB over a projection of 10 years.

To our knowledge, this is the first study that comprehensively evaluated the cost-effectiveness of MS/MS in low- and middle-income countries (LMICs). All previous economic evaluations of MS/MS were conducted in high-income countries. Of those evaluations, most of studies represented cost-utility analyses [14, 16, 17, 20, 22, 56] followed by cost-effectiveness analyses [15, 18, 19, 42, 57]. Most studies also adopted a societal perspective [14, 15, 19, 56] or used a health care provider’s perspective [17, 19, 20, 42], while a few applied the purchaser view [16, 22]. The most frequently used comparator in the studies is the no screening programme [14, 16–18, 20], followed by offering only PKU using the Guthrie test [15, 19, 42]. The result found in this study is not comparable with studies in other settings where most of the studies concluded that the screening programme is cost-effective either in screening for a combination diseases like in the US (California [16, 20], Wisconsin [17], Texas [22]), Australia [18], and UK [42] or screening for only some preferred disease like in UK [19] Canada [15]. As such, there are several differences between our study and the previous studies which need to be addressed.

Firstly, one important factor highly sensitive to the cost-effectiveness results of the screening programme is the incidence of diseases [14, 17]. A high burden in some particular diseases tends to make the intervention favourable, especially for diseases with effective outcomes of treatment such as PKU and medium-chain acyl-coenzyme A dehydrogenase deficiency (MCAD) [17, 19]; all previous studies were conducted in countries with a high incidence of PKU. For example, in the UK, the incidence of PKU used in that study was 9.00 cases per 100,000 live births while the incidence of MCAD claimed in the UK and American studies were 8.00 and 4.50 per 100,000 live births [17, 19], respectively. In contrast, the incidence of PKU adopted in our study was 2.22 in 100,000 live births or about four times lower than the UK incidence. Furthermore, MCAD was not included due to it being uncommon in Thai and Asian population [58]. In relation to this point, a systematic literature review of the economic evaluation of an MS/MS screening programme also mentioned that the dissimilarity in the demography of the countries and regions led to different MS/MS cost-effectiveness results [57].

Secondly, the cost of treatment is another key variable influencing the difference in findings. Ideally, an early diagnosis will prevent patients from serious clinical consequences resulting in less health resources required for treatment. If this is the case, it will lead to a programme that is more economically favourable. For example, in US, Schoen et al 2002 [20] indicated that if glutaric aciduria type I (GA1) patients were diagnosed early, the cost of treatment will be decreased about 46% compared with patients who were diagnosed late. The same amount of reduction was applied with other diseases such as MMA and PA [20]. In contrast, based on the treatment costs analysed from the hospitals, we did not find a difference between the costs of patients diagnosed early or late. This could be because even though early detection can prevent patients from severe clinical consequences at an acute period and help avoid some unnecessary costs for treatment, patients will still need more for preventive treatment in order to maintain their health. Therefore, patients diagnosed earlier can live longer and require more expensive treatment, some of which are required for a lifetime. This is agreeable with a previous study by Pandor et al 2004. [19] in the UK which found that there was no difference in treatment costs between the early diagnosis group and late diagnosis group for GA type I. This could be because the nature of the disease is extremely severe even though it was detected and treated early. Moreover, the effective treatment for this disease was not yet available. We believe that unless there is strong evidence, the assumption that early detection can reduce the cost of treatment should not be held.

Thirdly, there were huge differences in the outcome measurement, particularly the life-years gained between the early diagnosis and late diagnosis group. In a study by Schoen et al 2002 [20], the assumption of life-years gained of 20 years is added into the early detection group [20]. In an Australian study, it is assumed that patients who lived until 4 years of age could live up to the age of 66.2 [18]. Accordingly, those values used might be key factors which supported the favour of screening. However, a more conservative option was applied in a Canadian study where clinical data and the assumption about life expectancy were both used in the analysis [15]. In this case, it found that the average life-year gained was about 11 years (ranging from 4–25 years). In our analysis, using Markov modelling to follow the patients for lifetime estimated that having a screening programme will yield an average life-year gained of 13 years (ranging 4–40 years without outcome discounting). In particular, if the life expectancy gained by PKU patients was excluded, the average life-year gained for the rest of the IEM patients was only 8 years. We believe that from the scarcity of evidence, a conservative assumption for life expectancy should be the better option to apply to the model.

Lastly, the patient’s quality of life is one of most sensitive parameters to the result. The higher the quality of life achieved by patients diagnosed early, the more favourable screening became. From reviewing previous studies, we found that a relatively high value of health utility weight was applied to the studies which concluded that interventions were cost-effective. The utility weight of asymptomatic patients of 0.90 is used in the models by Feuchtbaum et al 2006 [16] as well as Tiwana et al 2012 [22], and 0.92 is used in the model by Autti-Roma et al 2005 [56] conducted in California and Texas in the US and Finland, respectively. It is important to note that those data were obtained from expert opinion [22, 56] or even research assumption [16] without reporting or mentioning the method of eliciting the utility weight of the patients. In our study, a more systematic approach was used for an analysis even though we had a small number of patients and most of them had mental retardation which resulted in difficulties in trying to extract utility weight data. As suggested by the Thai guidelines for conduction economic evaluations [59], the utility data was elicited by a proxy which is the group of people with knowledge of the diseases through the application of Indirectly measured utility methods (EQ-5D). This systematic tool for collecting the data was unique in that it can reduce the bias of the value of quality of life used [60, 61]. From the expert panel, we found that the utility of asymptomatic patients ranged from 0.49–0.84 for the six selected diseases (mostly under 0.71, only MCD was higher at 0.84). Obviously, the health utility weight applied for our analysis is lower than those of other studies which might plausibly result in the unfavourable outcome of the new intervention.

To argue about whether or not the number of diseases added into the analysis affected the results of study, we primarily believe that due to the variation of diseases, particularly in the incidence, level of severity, effective treatment, and costs of treatment, an individual assessment of the diseases is needed. The theory that including more diseases into the analysis would lead to favourable results really depends on whether the incremental benefit of the added diseases is above its incremental cost. If not, the added diseases can create a burden of costs for the whole screening programme. Thus, it can be concluded that it is not necessary to include all of the diseases into the model but instead give priority to evidence-based prioritised diseases and more attention to the details of each individual disease such as the effectiveness of treatment and relevant costs. The studies in Wisconsin [17] and the UK [19] proved that even screening for one or two diseases instead of a combination of 30 diseases may provide for a cost-effective intervention. In the same way, it does not mean that if the analysis indicates one or more of the diseases are economical for screening, another can be added to the programme and still be justified as cost-effective for the reason that there is no additional cost for screening. This is because the variable costs that come attached to expensive treatments can distort the results of study. By holding an expert panel, we believe that we have included the most significant diseases into the analysis, and that adding more diseases will not result in a more economical result unless there is new evidence such as high incidence of the diseases and effectiveness of treatment.

This study showed a zero utility weight (the equivalent of death) in three groups of patients including IVA, MSUD with neurological complications, PA with neurological and cardiomyopathy complications. This result is in line with a previous study which showed that Thai patients with a mental retardation combined with a complication, have a very low health utility that is close to zero [62]. For the three groups of patients, the zero utility can occur because the diseases are extremely severe. It is also important to address that apart from the main complications used as health states in the model, patients can also have other complications. In addition, combining these factors with the algorithm of the Thai EQ-5D could also explain the low score of patients. The observational study estimated a tariff-a coefficient that was used to subtract full health utility weight (1.00). It was found that in Thailand the tariff is high compared to other countries (i.e. UK and Japan) [63, 64]. For example, a tariff of constant term is valued -0.202 in Thailand and -0.081 in the UK. As for the utility score for state 33222, the calculated utility weight is -0.039 in Thailand compared with 0.161 in the UK.

The budget impact analysis points out that if the screening programme is implemented, the national healthcare payer must prepare a budget of at least 200 million THB each year. That amount can be considered substantial because it is comparable to almost one-tenth of the total healthcare budget allocated to all current screening activities financed by public sources in Thailand [65]. Therefore, implementing this screening seems to be very challenging under the rationale of affordability, a core concept of budget impact analysis. Another important point is that while the annual expenses of screening does not change much each year—thereby reflecting the relatively stable trend of population growth in Thailand—the treatment expenses is expected to rise sharply and will comprise a significant part of the entire budget in the future. This reveals that there are higher numbers of cumulative patients each year, each of which require lifelong treatment. This can be a crucial message delivered to policy makers when considering all aspects of providing the programme.

There are some limitations regarding the availability of data used in the model. Firstly, most of the incidences adopted in the analysis were foreign data of Asian countries. Nevertheless, the studies [6–8] indicated a similarity between uncommon IEM in Asia. So based on our current knowledge, the incidence should not be much different as Thailand is comparable with those countries in terms of ethnicity.

Secondly, although this study had advantages in using actual patient data to estimate the baseline clinical data, the information on the health outcomes of early-diagnosed patients still rely on unsubstantial evidence. Without existing well-established studies to observe the potential benefits of MS/MS screening for IEM, this study had to use information from observational studies that consisted of a small number of cases as well as expert opinion. However, we believe that we performed an extensive search in order to seek for the most credible evidence and used very conservative assumptions where information was limited.

Thirdly, the estimated cost of the screening programme did not include the cost of setting up a new screening unit to perform IEM screening and confirmation, transportation, and other logistics costs. Other potential expenses were also not included, example, human resources training such as the training of IEM specialists and related paramedics/metabolic dieticians/metabolic nurses. Nevertheless, we believe that if a new screening programme was provided, it is likely to be a part of the existing newborn screening institute in Thailand. Thus, it might not require lots of resource to set up a new centre for the screening programme.

Fourthly, the health utility weight was elicited from expert opinion. There were also many challenging issues about using QALY measurements in infants and children [66–68]. Nevertheless, given the lack of incidence and extreme difficulties of eliciting health from babies or patients with mental retardation, we believe that using an expert panel is a viable substitution.

Lastly, while there are several methods to measure health-related quality of life, based on the pros and cons of each method, the EQ-5D was selected as the most appropriate method for eliciting quality of life for economic evaluation by the Thai guidelines and this lead to the establishment of the national EQ-5D tariffs [69]. However, there are issues that needs to be addressed when using the EQ-5D, especially when it is used to estimate the utility of IEM patients. There are concerns for the generic health utility measures, for instance, the EQ-5D may not be sufficiently sensitive for people with mental health problems [70]. It is also possible that the EQ-5D does not assess some key health-related quality of life domains such as peer relations or family functioning [60]. In addition, as specific health problems of a certain disease may not be captured, it is possible to overestimate the utility weight which can potentially hamper the estimation of the incremental QALYs especially if the differences in effectiveness between comparators are marginal [70].

## Conclusions

In conclusion, the results of the study indicate that screening for inborn errors of metabolism in Thailand using MS/MS is economically unattractive given the threshold of cost-effectiveness in Thailand. Continuing the current screening programme as well as prioritising treatment for MCD, PKU, MSUD, and IVA patients diagnosed early is the appropriate action to take in order to deal with IEM. The budget impact analysis suggests that implementing the screening programme will incur considerable expenses. In addition, a nation-wide epidemiological study on the incidence of IEM in Thailand was strongly recommended to understand more about the magnitude of the diseases. Thus, we recommend that Thailand should perform a large-scale pilot study for an IEM screening programme as a further study.

## Supporting Information

S1 Table Yearly probability of death from other causes. (DOCX)

S2 Table Probability of admission as inpatient and average number of visit of outpatient per year. (DOCX)

S3 Table Difference of lifetime health outcomes and costs per-patient after early detection or late detection (without discounting). (DOCX)

S1 Search Strategy Search strategy. (DOCX)
