# Supplementary material for: An Economic Evaluation of Neonatal Screening for Inborn Errors of Metabolism Using Tandem Mass Spectrometry in Thailand
Source: PLoS One. 2015 Aug 10;10(8):e0134782. doi: 10.1371/journal.pone.0134782 (PMC4530882; doi:10.1371/journal.pone.0134782)
Supplement: S1 Table — (DOCX) [file pone.0134782.s001.docx]

| **Yearly probability of death from other causes** | **Distribution** | **Mean** | **SE** | **Reference** |
| --- | --- | --- | --- | --- |
| Age < 1 year | Beta | 8.08ˣ10-3 |  | [[1](#_ENREF_1)] |
| Age 1 to < 5 years | Beta | 9.50ˣ10^-4^ |  |  |
| Age 5 to < 10 years | Beta | 5.84ˣ10^-4^ |  |  |
| Age 10 to < 15 years | Beta | 5.38ˣ10^-4^ |  |  |
| Age 15 to < 20 years | Beta | 1.47ˣ10^-3^ |  |  |
| Age 20 to < 25 years | Beta | 1.76ˣ10^-3^ |  |  |
| Age 25 to < 30 years | Beta | 2.50ˣ10^-3^ |  |  |
| Age 30 to < 35 years | Beta | 3.22ˣ10^-3^ |  |  |
| Age 35 to < 40 years | Beta | 3.47ˣ10^-3^ |  |  |
| Age 40 to < 45 years | Beta | 4.07ˣ10^-3^ |  |  |
| Age 45 to < 50 years | Beta | 5.18ˣ10^-3^ |  |  |
| Age 50 to < 55 years | Beta | 7.25ˣ10^-3^ |  |  |
| Age 55 to < 60 years | Beta | 1.04ˣ10^-2^ |  |  |
| Age 60 to < 65 years | Beta | 1.42ˣ10^-2^ |  |  |
| Age 65 to < 70 years | Beta | 2.08ˣ10^-2^ |  |  |
| Age 70 to < 75 years | Beta | 3.16ˣ10^-2^ |  |  |
| Age 75 to < 80 years | Beta | 4.95ˣ10^-2^ |  |  |

**Table S1** Yearly probability of death from other causes

**Reference**

1. Bundhamcharoen K, Teerawattananon Y, Vos T, Begg S (2002) Burden of disease and injuries in Thailand: priority setting for policy. Bangkok.
