# Supplementary material for: An Economic Evaluation of Neonatal Screening for Inborn Errors of Metabolism Using Tandem Mass Spectrometry in Thailand
Source: PLoS One. 2015 Aug 10;10(8):e0134782. doi: 10.1371/journal.pone.0134782 (PMC4530882; doi:10.1371/journal.pone.0134782)
Supplement: S2 Table — (DOCX) [file pone.0134782.s002.docx]

**Table S2** Probability of admission as inpatient and average number of visit of outpatient per year

| **Probability of admission as inpatient per year** |  |  |  |  |
| --- | --- | --- | --- | --- |
| First year of treatment |  |  |  |  |
| PKU | Beta | 0.18 | 0.15 | Hospital database |
| IVA | Beta | 1.00 | 0.00 | Hospital database |
| MMA | Beta | 1.00 | 0.00 | Hospital database |
| PA | Beta | 1.00 | 0.00 | Hospital database |
| MSUD | Beta | 1.00 | 0.00 | Hospital database |
| MCD | Beta | 0.76 | 0.00 | Hospital database |
| Second year of treatment onward |  |  |  |  |
| IVA | Beta | 0.30 | 0.21 | Hospital database |
| MMA | Beta | 0.71 | 0.21 | Hospital database |
| PA | Beta | 0.88 | 0.11 | Hospital database |
| MSUD | Beta | 0.61 | 0.24 | Hospital database |
| MCD | Beta | 0.29 | 0.21 | Hospital database |
|  |  |  |  |  |
| **Average number of visit of outpatient per year** |  |  |  |  |
| PKU | Gamma | 4.84 | 0.58 | Hospital database |
| IVA | Gamma | 7.17 | 7.17 | Hospital database |
| MMA | Gamma | 10.52 | 0.88 | Hospital database |
| PA | Gamma | 10.75 | 0.89 | Hospital database |
| MSUD | Gamma | 6.65 | 0.85 | Hospital database |
| MCD | Gamma | 2.3 | 0.52 | Hospital database |
| **Hospital outpatient care cost per time** |  |  |  |  |
| PKU | Gamma | 3,380 | 555 | Hospital database |
| IVA | Gamma | 6,264 | 1,121 | Hospital database |
| MMA | Gamma | 10,332 | 2,570 | Hospital database |
| PA | Gamma | 5,708 | 2,402 | Hospital database |
| MSUD | Gamma | 2,697 | 612 | Hospital database |
| MCD | Gamma | 1,789 | 695 | Hospital database |
