# Supplementary material for: An Economic Evaluation of Neonatal Screening for Inborn Errors of Metabolism Using Tandem Mass Spectrometry in Thailand
Source: PLoS One. 2015 Aug 10;10(8):e0134782. doi: 10.1371/journal.pone.0134782 (PMC4530882; doi:10.1371/journal.pone.0134782)
Supplement: S3 Table — (DOCX) [file pone.0134782.s003.docx]

**Table S3** Difference of lifetime health outcomes and costs per-patient after early detection or late detection (without discounting)

| **Disease** | **Cost** | | |  | **Life years** | | |  | **QALY** | | |
| --- | --- | --- | --- | --- | --- | --- | --- | --- | --- | --- | --- |
|  | **Diagnosis** | | **difference** |  | **Diagnosis** | | **difference** |  | **Diagnosis** | | **difference** |
|  | **Early** | **Late** |  |  | **Early** | **Late** |  |  | **Early** | **Late** |  |
| **PKU** | 7,608,484 | 4,395,672 | 3,212,812 |  | 73.32 | 32.96 | 40.36 |  | 51.91 | 18.92 | 32.99 |
| **IVA** | 8,298,487 | 7,000,223 | 1,298,264 |  | 39.19 | 30.62 | 8.57 |  | 25.84 | 16.50 | 9.34 |
| **MMA** | 15,788,380 | 11,123,478 | 4,664,902 |  | 33.24 | 22.67 | 10.57 |  | 17.94 | 11.07 | 6.88 |
| **PA** | 5,448,421 | 3,232,267 | 2,216,153 |  | 12.64 | 7.14 | 5.50 |  | 2.10 | 0.82 | 1.28 |
| **MSUD** | 5,917,971 | 5,164,562 | 753,409 |  | 26.16 | 20.36 | 5.80 |  | 6.49 | 1.24 | 5.25 |
| **MCD** | 6,247,411 | 6,542,110 | -294,699 |  | 73.48 | 69.48 | 4.00 |  | 61.46 | 56.22 | 5.24 |
