# Supplementary material for: An Economic Evaluation of Neonatal Screening for Inborn Errors of Metabolism Using Tandem Mass Spectrometry in Thailand
Source: PLoS One. 2015 Aug 10;10(8):e0134782. doi: 10.1371/journal.pone.0134782 (PMC4530882; doi:10.1371/journal.pone.0134782)
Supplement: S1 Search Strategy — (DOCX) [file pone.0134782.s004.docx]

**Supporting information 4**

Database: Embase, Ovid MEDLINE(R)

Search period 1970-2012

Search Strategy:

--------------------------------------------------------------------------------

1 inborn errors of metabolism.mp. [mp=ti, ab, hw, tn, ot, dm, mf, dv, kw, nm, kf, px, rx, an, ui] (4553)

2 phenylketonur$.mp. [mp=ti, ab, hw, tn, ot, dm, mf, dv, kw, nm, kf, px, rx, an, ui] (11333)

3 isovaleric acidemia.mp. [mp=ti, ab, hw, tn, ot, dm, mf, dv, kw, nm, kf, px, rx, an, ui] (301)

4 isovaleric aciduria.mp. [mp=ti, ab, hw, tn, ot, dm, mf, dv, kw, nm, kf, px, rx, an, ui] (42)

5 isovaleric acidaemia.mp. [mp=ti, ab, hw, tn, ot, dm, mf, dv, kw, nm, kf, px, rx, an, ui] (68)

6 3 or 4 or 5 (401)

7 methylmalonic acidemia.mp. [mp=ti, ab, hw, tn, ot, dm, mf, dv, kw, nm, kf, px, rx, an, ui] (1154)

8 methylmalonic aciduria.mp. [mp=ti, ab, hw, tn, ot, dm, mf, dv, kw, nm, kf, px, rx, an, ui] (1077)

9 methylmalonic acidaemia.mp. [mp=ti, ab, hw, tn, ot, dm, mf, dv, kw, nm, kf, px, rx, an, ui] (180)

10 7 or 8 or 9 (2231)

11 propionic acidemia.mp. [mp=ti, ab, hw, tn, ot, dm, mf, dv, kw, nm, kf, px, rx, an, ui] (1021)

12 propionic aciduria.mp. [mp=ti, ab, hw, tn, ot, dm, mf, dv, kw, nm, kf, px, rx, an, ui] (85)

13 propionic acidaemia.mp. [mp=ti, ab, hw, tn, ot, dm, mf, dv, kw, nm, kf, px, rx, an, ui] (220)

14 11 or 12 or 13 (1207)

15 maple syrup urine disease$.mp. [mp=ti, ab, hw, tn, ot, dm, mf, dv, kw, nm, kf, px, rx, an, ui] (2091)

16 multiple carboxylase deficienc$.mp. [mp=ti, ab, hw, tn, ot, dm, mf, dv, kw, nm, kf, px, rx, an, ui] (261)

17 1 or 2 or 6 or 10 or 14 or 15 or 16 (19806)

18 Neonatal Screening.mp. [mp=ti, ab, hw, tn, ot, dm, mf, dv, kw, nm, kf, px, rx, an, ui] (11567)

19 17 and 18 (1318)

20 19 and 1970:2012.(sa_year). (1190)

21 remove duplicates from 20 (975)

22 from 20 keep 1-1190 (1190)

23 remove duplicates from 22 (975)

**Inclusion criteria**

1. study design: all study types

2. subjects: neonatal or newborn infants

3. intervention: tandem mass spectrometry

4. outcome: report the outcome of screening and without screening groups

**Exclusion criteria**

1. papers published before 1970 (using filter of the database)

2. any papers not available in the English language

3. report outcome only screening or without screening group

**Included literature**

| **No.** | **Literature** |  |
| --- | --- | --- |
| 1 | Dionisi-Vici C, Deodato F, Roschinger W, Rhead W, Wilcken B (2006) 'Classical' organic acidurias, propionic aciduria, methylmalonic aciduria and isovaleric aciduria: long-term outcome and effects of expanded newborn screening using tandem mass spectrometry. J Inherit Metab Dis 29: 383-389 |  |
| 2 | Gonzalez MJ, Gutierrez AP, Gassio R, Fuste ME, Vilaseca MA, et al. (2011) Neurological complications and behavioral problems in patients with phenylketonuria in a follow-up unit. Mol Genet Metab 104 Suppl: S73-79 |  |
| 3 | Grunert SC, Mullerleile S, de Silva L, Barth M, Walter M, et al. (2012) Propionic acidemia: neonatal versus selective metabolic screening. J Inherit Metab Dis 35: 41-49 |  |
| 4 | Morton DH, Strauss KA, Robinson DL, Puffenberger EG, Kelley RI (2002) Diagnosis and treatment of maple syrup disease: a study of 36 patients. Pediatrics 109: 999-1008. |  |
| 5 | Fisch RO, Bilek MK, Bruhl HH (1976) Causes of death of institutionalized phenylketonuric (PKU) patients-A national survey. Minn Med 59: 306-309 | Reference searching |
| 6 | Dobson JC, Williamson ML, Azen C, Koch R (1977) Intellectual assessment of 111 four-year-old children with phenylketonuria. Pediatrics 60: 822-827 | Reference searching |
| 7 | Grunert SC, Wendel U, Lindner M, Leichsenring M, Schwab KO, et al. (2012) Clinical and neurocognitive outcome in symptomatic isovaleric acidemia. Orphanet J Rare Dis 7: 9 | Reference searching |
| 8 | Aoki K, Wada Y (1988) Outcome of the patients detected by newborn screening in Japan. Acta Paediatr Jpn 30: 429-434 | Reference searching |
